# Fault monitoring method of domestic waste incineration slag sorting device based on back propagation neural network

**DOI:** 10.1016/j.heliyon.2024.e27396

**Published:** 2024-03-04

**Authors:** Hao Xu, Dongdong Huan, Jihong Lin

**Affiliations:** a School of Mechanical and Electrical Engineering, Soochow University, Suzhou, 215137, China; b School of Ecological Environment and Urban Construction, Fujian University of Technology, Fuzhou, 350118, China

**Keywords:** Back propagation neural network, Domestic waste, Sorting device, Fault diagnosis, Monitoring model, Accuracy

## Abstract

The main monitoring points of traditional sorting equipment fault monitoring methods are usually limited to the inlet and outlet, making it difficult to monitor the internal equipment, which may affect the accuracy of fault monitoring. Therefore, a new fault monitoring method based on back propagation neural network has been studied and designed, which is mainly applied to the sorting device of domestic waste incineration slag. The fault monitoring modeling variables of the domestic waste incineration slag sorting device are selected to determine the operation status of the sorting device. Based on back propagation neural network, a fault monitoring model for the sorting device of municipal solid waste incinerator slag is constructed, and the fault data of the sorting device is trained in the model, so that the fault data of the sorting device can be optimized faster, thus improving the accuracy of fault monitoring. Through comparative experiments with traditional methods, it has been confirmed that this fault monitoring method based on back propagation neural network has significant advantages in detection performance, demonstrating its potential in practical applications.

## 1 Introduction

Domestic waste has different forms, regular, irregular, colorful, different shapes and colors, and different materials. Similar plastic, metal, paper, glass and other waste emerge in endlessly. The waste is not clean, there are different degrees of dirt, and there is also the problem of crushing damage [1]. Various consumer products in daily life, such as disposable products such as mineral water bottles, plastic bags, and toilet paper, will become waste with various shapes and stains after use [2]. In the current field of urban management and environmental protection, the disposal of domestic waste has become an urgent problem to be solved. If waste is not handled properly, it may form a "waste siege" phenomenon, posing a threat to the urban environment and residents' health. Faced with a large amount of waste, waste disposal is required. Among them, domestic waste incineration is a widely used treatment method, which can not only effectively reduce the volume of waste, but also recover energy [3,4]. However, the slag produced during the incineration process contains a large amount of inorganic and metallic substances, which requires effective sorting and treatment. The waste incinerator is arranged according to the amount of waste treatment, and the grate is generally used for garbage treatment in the drying section, combustion section and burn out section. The management and monitoring of imports, furnaces, and exports directly affect processing efficiency [5]. The domestic waste incineration slag sorting device plays a key role in this process, but due to the complexity of its operating environment, the risk of failure is relatively high. Therefore, effective fault monitoring and prevention of these devices is key to ensuring their stable operation. Traditional fault monitoring methods mostly rely on manual detection or simple mechanical sensors. These methods have obvious limitations in processing complex data and achieving early fault warning. With the development of artificial intelligence technology, especially the application of neural networks in the field of pattern recognition and prediction, new possibilities are provided for improving fault monitoring methods of slag sorting devices. The Back Propagation (BP) network can approximate any nonlinear function with arbitrary accuracy, which makes it the most widely used artificial intelligence algorithm at present. Many scholars have analyzed deep learning algorithms and waste incineration. To effectively predict and control the amount of nitrogen oxides emitted by cities, Meng et al. proposed a topological modular technology inspired by biology. In the process, an adaptive task-oriented radial basis function neural network was used to construct different modules, which lays the foundation for the generalization ability of the model. The results showed that the proposed model can have high running speed and accuracy [6]. To effectively predict the heat energy generated by waste incineration, researchers such as Yatim et al. proposed a prediction method based on artificial neural networks (ANNs) and multiple linear regression. The experiment converted waste into energy to develop new energy sources and estimated the high calorific value of biomass waste. The results showed that this method could accurately predict the heat energy generated by waste incineration [7]. To achieve online optimization of boiler operation, Ye et al. proposed a new method based on information integration and case reasoning. The experiment combined offline data and online combustion data by using the constructed framework, and verified the superiority of the proposed method through simulation experiments. The results showed that the combustion efficiency of the boiler increased by 0.31%, and the maximum production of nitrogen oxides was significantly reduced by 41.22mg/Nm3. This showed that the proposed method could be used for online real-time combustion monitoring [8]. To predict the energy generated by garbage burning, Ma et al. proposed an energy prediction method based on big data and ANNs. The experiment analyzed data from more than 400 factories in China and found that environmental footprint had a significant negative correlation with social economy. The constructed model could be used to effectively monitor urban exhaust gas emissions, with monitoring errors ranging from 0.003 to 0.09 [9].

At the same time, some scholars have also discussed waste management and treatment methods. To predict solid waste management problems, Xi et al. proposed a waste separation model based on ANN and analytic hierarchy process. The experiment predicted missing indicators by establishing a method based on scanning optimization and machine learning. The results showed that the method proposed in the experiment provided a new way for solid waste separation and could accurately separate waste [10]. To reduce the impact of greenhouse gases, scholars such as Fallah et al. proposed a landfill gas cleaning method based on multi-level neural networks. The experiment first preprocessed the data and evaluated independent and important weather as input variables to provide a suitable data set to the model. The results showed that this method had high prediction performance, and the mean absolute percentage error obtained was only 3.03% [11]. To effectively process waste, scholars such as Feng et al. proposed a waste disposal model based on deep learning. At the same time, to effectively process waste images, a data set containing multiple waste forms was established. The results showed that the proposed method could detect waste with an accuracy of 93%, which could greatly promote the development of automated production [12]. To reform the management of urban waste, Wang et al. proposed a waste classification method based on deep learning classifiers and cloud computing technology. At the same time, to facilitate waste disposal, the types of recyclable waste were subdivided. The results showed that the proposed method had the highest accuracy in waste detection, and the system had a short running time [13].

Overall, relatively extensive research has been done on applying deep learning and various intelligent algorithms to waste management and control. These studies not only improve the operating efficiency and safety of the incineration slag sorting device, but also provide valuable experience and technical support for the development of intelligent waste treatment systems. However, many challenges and opportunities still exist, such as algorithm optimization, data processing capability improvement, and application adaptability under different operating conditions. In view of this, the experiment proposes a fault monitoring method for the domestic waste incineration slag sorting device based on an improved BP neural network. It is expected that the powerful data processing and learning capabilities of the BP neural network can effectively identify and predict potential faults of the slag sorting device. This enables more accurate and timely fault monitoring to support the overall situation of sustainable urban development and environmental protection.

The structure of the article can be mainly divided into 4 parts. Part 1 is the introduction. The research background, importance and purpose of the research are introduced, and the current research status and existing problems in the field of domestic waste incineration slag sorting at home and abroad are briefly described. Part 2 is the method. The proposed fault monitoring method based on BP neural network is described in detail, including network structure, data preprocessing, feature extraction and network training process. Part 3 is the analysis of experimental results, and comparative analysis of the performance of the proposed method and other existing technologies to verify the effectiveness of the method. Section 4 is the conclusion and future work. It summarizes the research results, puts forward conclusions, and looks forward to future research directions.

## 2 Design of fault monitoring method for domestic waste incineration slag sorting device based on BP neural network

### 2.1 Select fault monitoring modeling variables of domestic waste incineration slag sorting device

In the process of waste incineration, the sorting device is mainly composed of feeding, distribution, slag discharge from the primary combustion chamber, secondary combustion chamber, air supply, induced air, monitoring instruments and other devices. The hopper transports the waste slag from the storage pit to the feeding port of the sorting device. After the waste double pushing device is pushed into the sorting device, it is evenly sent through the distribution to the sorting unit of the matching device for sorting [14,15]. The main process parameters of incineration slag sorting device are shown in Table 1 below.

**Table 1:** Main process parameters of incinerator slag sorting device.

classification	parameter	classification	parameter
Sorting capacity	Normal sorting capacity 90 t/d, maximum sorting capacity 110 t/d	Air distribution	Bottom wind: 12∼18Hz, waist wind opening: 30% distribution, induced wind: 20∼30Hz configuration
Lower calorific value of waste	3300∼5300 kJ/kg，Water content≤50%	Negative pressure value of primary combustion chamber	−50Pã100Pa
Primary combustion chamber furnace temperature	750 °C–950 °C	Discharge speed	Speedometer 200 rpm–300rom optional
Secondary combustion chamber furnace temperature	850 °C–1100 °C	Furnace grate speed	100 rpm/Hours∼600 rpm/Hours
Residence time of flue gas in secondary combustion chamber	≥2s	Secondary air supply frequency	0Hz–40Hz
Feed quantity	Correction based on 4 t/h	Excess air coefficient	1.5

**Fig. 1:**
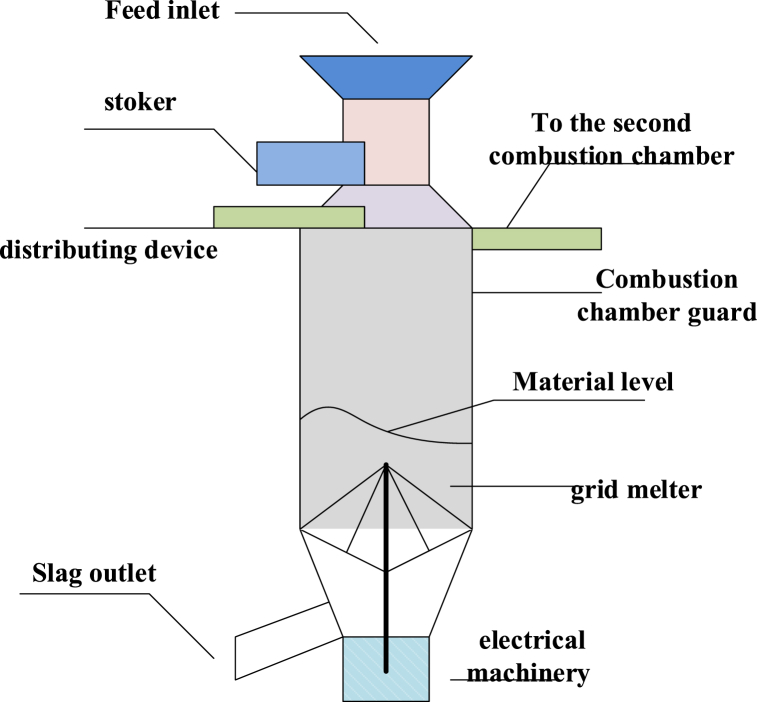
Schematic diagram of incinerator sorting device.

As shown in Table 1, before falling into the sorting furnace, the slag will be evenly sent into the furnace of the sorting combustion chamber through the distribution device. In the furnace of the first combustion chamber, the continuously fed slag will form a material layer on which the slag is mainly sorted. Slag is continuously input to maintain sorting on the material layer [16]. The sorted slag is broken through the grate, forming ash and falling to the slag outlet for discharge. The slag sorting air supply of the primary combustion chamber is mainly primary air, which enters the furnace from below the grate and passes through the sorting material layer upwards. Before reaching the material layer, the primary air will be pre-sorted in the furnace, so as not to affect the sorting temperature near the material layer. When the primary air reaches the material layer, the oxygen in it reacts with the combustible substances in the slag to sort the slag [17]. In the sorting process, due to the different density of the slag in the material layer, the oxygen supply for sorting is uneven. In this paper, the secondary air enters the primary combustion chamber from the top of the sorting material layer to ensure that the slag is completely sorted. The incinerator sorting device is shown in Fig. 1 below.

As shown in Fig. 1, during the operation of the sorting device, faults such as partial burn through, un-smooth slag discharge and coking often occur. According to the fault status data, this paper selects the corresponding data signal as the fault modeling variable of the sorting device [18], including sorting capacity under normal and fault operating conditions, low waste calorific value, main and secondary combustion chamber furnace temperature, air distribution, main combustion chamber negative pressure, discharge speed, grate speed, and excess air coefficient. The sorting data under normal operation status is selected as the original data signal. The normal operation data is divided into an average temperature difference range, and the data beyond or below this range is the fault data, so as to ensure the variable monitoring effect. In addition, the study also noted some parameters with strong correlation and importance that significantly deviate from the normal range under fault conditions, such as the negative pressure and temperature of the main and the secondary combustion chambers. Furthermore, although these parameters may fluctuate within a certain range under normal operating conditions, significant abnormal fluctuations or sustained deviations from normal values may occur under fault conditions, providing important clues for identifying and predicting faults.

### 2.2 Building fault monitoring model of domestic waste incineration slag sorting device based on BP neural network

To realize the effective monitoring of sorting device faults, this paper uses BP neural network to monitor sorting device faults. In the whole process of slag sorting, the main indicator of sorting device fault diagnosis is device temperature. During the sorting of the sorting device, the temperature parameters in the device change significantly [19,20]. Neural networks with 3 or more layers can approach any nonlinear continuous function with arbitrary accuracy when the number of neurons is sufficient. In general, it is necessary to construct a neural network with the minimum number of network layers as much as possible, so this article selects a 3-layer BP network structure. A three-layer BP network can complete the mapping from N dimension to M dimension, and the BP network contains a hidden layer, which can determine the node dimensions of input layer and output layer. The number of hidden layer nodes is determined according to the hidden layer theorem, and the formula is as follows:

(1) Q=2×m+1

**Fig. 2:**
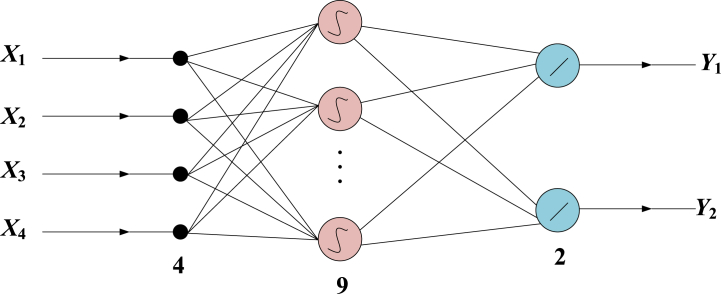
Schematic diagram of neural network subnet structure.

In formula (1), Q is the number of hidden layer nodes. m is the number of input layer nodes. The number of hidden layer nodes has a significant impact on the performance of BP networks [21,22]. At present, there is no ideal analytical formula for determining the number of neuron nodes, which is usually determined by combining empirical formulas and continuous experimentation. In the specific design, this article adopts the trial and error method, which compares and trains networks with different numbers of hidden layer neurons. The training frequency is set to 2000, and the target error is all 0 001, 10 comparative experiments were conducted on networks with [7,16] hidden layer nodes to determine the optimal parameters. Using the neural network structure, it sets the learning rate to 0.1, and the BP neural network structure is shown in Fig. 2 below.

As shown in Fig. 2, under the condition of this structure, the initial weight threshold of the fault monitoring model is set as a random number between [−0.1,0.1], and the hidden node is activated using the activation function. The activation function expression is as follows:

(2) $$y=f\left(m\right)=\frac{1}{1+e^{-\lambda }}$$

In formula (2), f(m) is the activation function expression. $e^{-\lambda }$ is a natural constant. λ is the function gain, which determines the slope of the function. In this paper, training data is used to train the neural network [23,24]. After the training, 10 categories of slag are sorted out, and two categories are obtained by adding recyclable slag and non recyclable slag. The cross entropy loss of the two categories is calculated. The formula is as follows:

(3) $$L=\varepsilon L\left(Y_{1},Y_{mat}\right)+\left(1-\varepsilon \right)L\left(Y_{2},Y_{rat}\right)$$

In formula (3), L is the cross entropy loss function. ε is the weight factor. $Y_{1}$, $Y_{2}$ are the output layer of neurons. $Y_{mat}$ is the probability vector of sorting fault data. $Y_{rat}$ is the corresponding one pot tag [25]. After neuron processing, the derivation of fault data of sorting device is as follows:

(4) $$\frac{\partial L}{\partial f\left(m\right)}=\varepsilon \frac{\partial L\left(Y_{1},Y_{mat}\right)}{\partial f\left(m\right)}+\left(1-\varepsilon \right)\frac{\partial L\left(Y_{2},Y_{rat}\right)}{\partial f\left(m\right)}$$

In formula (4), ∂ is derivative. $\frac{\partial L}{\partial f\left(m\right)}$ represents the probability value obtained from softmax layer.

(5) $$L\left(Y_{1},Y_{mat}\right)=-\sum_{i=1}^{M}Y_{i}\;\log\;P_{i}$$

In formula (5), $Y_{i}$ is the output layer of neurons. $P_{i}$ is the partial derivative. Assuming that the sorting property of i is the same as that of garbage, the fault monitoring model expression is:

(6) $$\frac{\partial L}{\partial P_{i}}=\left\{ \begin{array}{c} \left(1-\frac{1}{p}+\frac{\varepsilon }{p}\right)p_{i}-\varepsilon \\ \left(1-\frac{1}{p}+\frac{\varepsilon }{p}\right)p_{i}\\ p_{i} \end{array}\right.$$

In formula (6), $\frac{\partial L}{\partial P_{i}}$ is the fault monitoring model expression, α=1,2,⋯,p, p is the probability corresponding to the slag sorting property. $p_{i}$ is the probability corresponding to the i-th slag sorting property. When 0 ≤ ε ≤ 1, the 10 types of neurons have the same punishment for the sorting fault data of different labels, and the output of other neurons is 0. At this time, the fault monitoring accuracy is high, showing an increasing trend, which can ensure the fault monitoring effect of the sorting device [26,27]. For training samples $\left\{o_{s}^{\left(\alpha \right)},o_{s}^{\left(\alpha \right)}\right\}$, where s=1,2,⋯,m and m represents the number of output layer data, and p represents the number of samples. The determination of neural network parameters is carried out by finding the optimal solution to the equation shown in formula (7).

(7) $$\min_{\omega_{ij},c_{js}}E^{\left(k\right)}=\sum_{\alpha =1}^{p}\sum_{s=1}^{m}\left(o_{s}^{\left(\alpha ,k\right)}-o_{s}^{↔\left(\alpha \right)}\right)2\leq \varepsilon_{0}$$

In formula (7), k is the number of training sessions. $\omega_{ij}$ and $c_{js}$ are the connection weights from the input layer to the hidden layer, and the connection weights from the hidden layer to the output layer, respectively. $\varepsilon_{0}$ is the given error range. The study adopts the fastest descent method, where the weight changes along the negative gradient direction of the function error, so that the error converges to the minimum pointv [28,29]. At the same time, in order to accelerate the convergence process of network training, a BP algorithm with momentum factor is studied to gradually correct the network weights, as shown in formula (8).

(8) $$\left\{ \begin{array}{c} \omega_{ij}^{\left(k+1\right)}=\omega_{ij}^{\left(k\right)}+\Delta \omega_{ij}^{\left(k\right)}+\delta_{1}\left(\omega_{ij}^{\left(k\right)}-\omega_{ij}^{\left(k-1\right)}\right)\\ c_{js}^{\left(k+1\right)}=c_{js}^{\left(k\right)}+\Delta c_{js}^{\left(k\right)}+\delta_{2}\left(c_{ij}^{\left(k\right)}-c_{ij}^{\left(k-1\right)}\right) \end{array}\right.$$

### 2.3 Network training and prediction

**Fig. 3:**
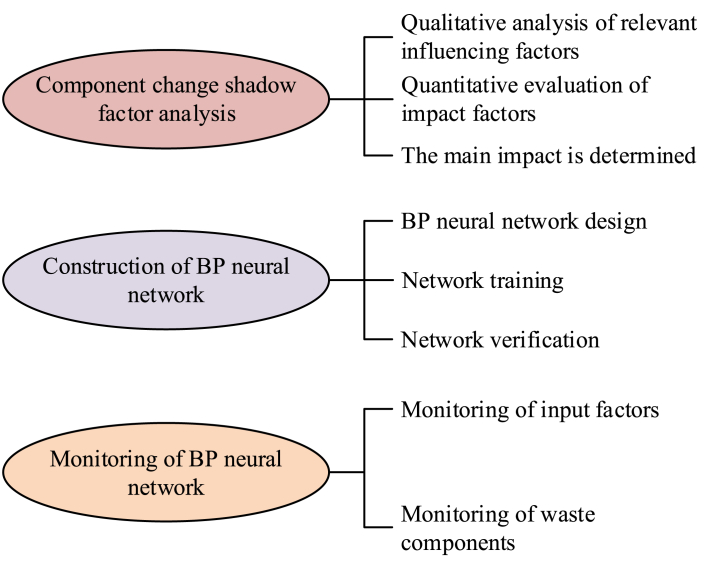
Construction of monitoring model of municipal solid waste component sorting device.

Network training refers to using the determined standard output layer data to perform continuous model calculations, determining calculation errors, and continuously adjusting and optimizing network weights and thresholds until the network calculation results meet the model calculation error range. On this basis, based on the network weights, thresholds, and necessary input data obtained through training, the BP neural network is used for model prediction [30]. Fig. 3 shows the technical route for establishing a prediction model for municipal solid waste components.

Based on previous research results and relevant work experience, it is believed that the factors affecting municipal solid waste incineration can be attributed to waste composition, moisture content, oxygen supply, furnace temperature and pressure, combustion technology, pollution control facilities, pre-treatment and separation, operation and maintenance, etc. Through the correlation analysis between the classification of domestic waste in a certain city and various influencing factors, it is found that among the above influencing factors, there are five factors that are highly correlated with the incineration and sorting of domestic waste: waste composition, moisture content, oxygen supply, furnace temperature and pressure, combustion technology and pollution control facilities [31,32]. Therefore, the above five factors can be used as the main input parameters of the BP neural network prediction model. At the same time, in the results section, the reliability and robustness of the model are evaluated by simulating fault scenarios and abnormal situations.

## 3 Experiment

To verify whether the fault monitoring method designed in this paper can be applied to real life, this paper carried out experimental analysis on the above methods. In the three fault states of the sorting device, the network was trained using the steepest descent method, momentum BP method, adaptive learning rate BP method, elastic BP method, Quasi-Newton method, and Levenberg-Marquard (LM) algorithm, respectively. Then, the fault monitoring method based on traditional household waste incineration slag sorting device was explored, as well as the monitoring effect of the BP neural network designed in this article on the fault monitoring of household waste incineration slag sorting device. The specific experimental preparation process and experimental results are shown below.

### 3.1 Experiment preparation

**Fig. 4:**
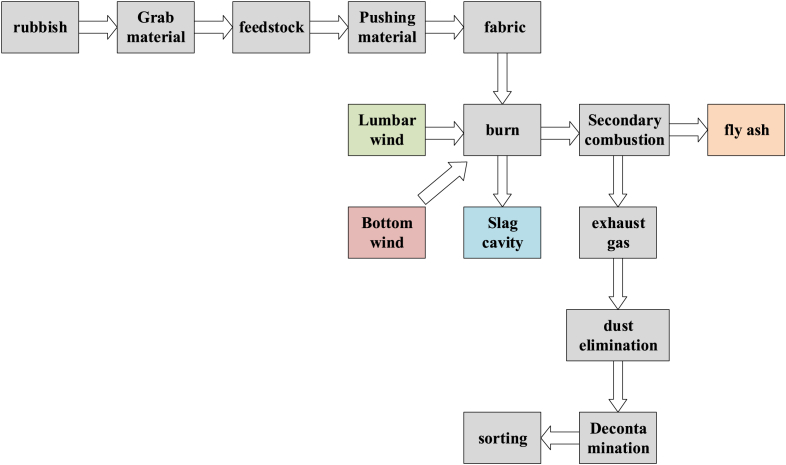
Domestic waste incineration slag sorting flow chart.

In the process of waste incineration, it is difficult to sort the incineration slag, and it is very easy to have faults. There is electronic waste, glass waste, hazardous waste, kitchen waste, metal waste, fabric waste, recyclable and non-recyclable paper waste, recyclable and non-recyclable plastic waste, etc. In public places, the generation probability of recyclable and non-recyclable waste is far higher than other waste, and the incineration slag sorting is more difficult, which affects the processing efficiency of the sorting device [33,34]. In this experiment, the BP neural network fault monitoring model was used to mark different types of household waste with different label neurons, so that the output of neurons with different material labels was 0 as far as possible. The output of neurons with the same material label was 1. Under the influence of the loss function, the sorting fault monitoring penalties for different neurons in the domestic waste were different, and the trend changed from increasing to decreasing, so as to avoid the monitoring model meeting the conditions prematurely, thus enhancing the fault monitoring accuracy. The sorting of domestic waste incineration slag is shown in Fig. 4 below.

**Fig. 5:**
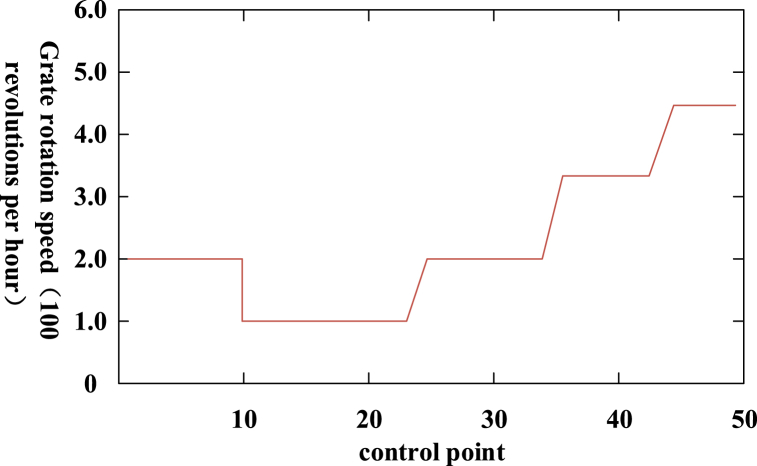
Control diagram of incinerator grid.

As shown in Fig. 4, after the domestic waste was incinerated in the incinerator, the slag was put into the sorting device switch for sorting. After the ballast was put in, the light of the sorting device was turned on to facilitate the sorting monitoring of the monitoring unit of the sorting device. After the waste and slag were delivered, the recyclable and non-recyclable waste on both sides shall be equipped with green and red indicator lights. After the slag sorting was completed, the corresponding indicator lights shall be turned on to ensure the safe operation of the sorting device. The control of incinerator grate is shown in Fig. 5 below.

As shown in Fig. 5, to eliminate the impact of the combustion state of the incinerator itself and ensure the effectiveness of this experiment, control points were set up at various locations on the grate of the incinerator after the household waste was put into the incinerator, and the operation effect of the control points was monitored. From Fig. 4, at the control points 10, 22, 34 and 41, the rotational speed of the grate has changed correspondingly, that is to say, the incineration path here was difficult to burn. The slag of the above control points was put directly into the sorting device, and the sorting device would sort the slag to ensure the slag sorting effect. It is known that the temperature of the slag sorting device will change during the sorting, which is similar to the temperature of the incinerator burning garbage. The higher the temperature of the sorting device, the more obvious the fault of the sorting device. The study screened parameter data for normal and faulty operation of the equipment from the operation logs and historical data of the sorting device, and randomly selected 10 sets of data based on the type of fault as the basis for subsequent fault diagnosis. Based on 10 sets of data for each type of fault, it obtained the maximum and minimum values of temperature, then the maximum value was increased by 30 °C and the minimum value was decreased by 30 °C to obtain the judgment range of the fault. The monitoring variables for normal and faulty operation of sorting equipment are shown in Table 2.

**Table 2:** Experimental data sheet.

Group	Localized burn through		Poor slag discharge		coking	
	Normal data/°C	Fault data/°C	Normal data/°C	Fault data/°C	Normal data/°C	Fault data/°C
Group 1	154.776	627.142	145.423	220.950	143.880	361.038
Group 2	182.630	616.525	131.048	228.533	130.450	360.817
Group 3	185.545	619.992	127.034	246.725	142.403	398.721
Group 4	183.710	499.633	136.477	223.657	165.288	402.025
Group 5	155.373	504.087	128.123	221.009	148.075	364.325
Group 6	139.190	531.696	118.561	206.998	124.488	399.750
Group 7	160.871	522.487	129.432	220.982	189.212	364.325
Group 8	170.092	501.146	145.789	225.579	148.4136	345.475
Group 9	171.104	500.312	132.087	210.677	164.225	332.153
Group 10	166.104	613.808	126.454	206.167	123.850	339.305

As shown in Table 2, the operation data of the incinerator slag sorting device was divided into 10 groups in this experiment. The faults of the sorting device were local burn through fault, un-smooth slag discharge fault, coking fault, etc, which are marked as LB, US, and CF respectively. The normal and the fault operation data were analyzed respectively to ensure a clear analysis of the fault data. During the incineration, the temperature of the incinerator directly affected the temperature of the sorting device. The average temperature, cooling temperature, temperature change rate, cooling change rate and other indicators of the incineration belt were all indicators that affect fault monitoring. In this paper, in the BP neural network fault monitoring model, the temperature data of the sorting device in normal operation was input. If the temperature exceeded the temperature and was lower than the temperature, the sorting device fault was determined to avoid the problem of fault monitoring failure.

### 3.2 Experimental results

The network was trained using the steepest descent method, momentum BP method, adaptive learning rate BP method, elastic BP method, Quasi-Newton method, and LM algorithm. The comparison of the training results is shown in Table 3.

**Table 3:** Training results.

No.	Method	Iterations	Error
1	Steepest descent	>2000	0.00225
2	Momentum BP	>2000	0.00372
3	Adaptive BP	221	0.00099
4	Quasi-Newton	6	0.00028
5	Levenberg-Marquard algorithm	7	0.00024
6	Elastic BP	5	0.00021
7	The method proposed in this article	3	0.00005

In Tables 3 and it can be observed that as the system iterated, the monitoring errors corresponding to the steepest descent method, momentum BP method, adaptive learning rate BP method, elastic BP method, Quasi-Newton method, and LM algorithm were 0.00225, 0.00372, 0.00099, 0.00028, 0.00024, and 0.00021, respectively. The steepest descent method and momentum BP method had more than 2000 iterations, but the elastic BP method had the least number of iterations. When the system iterated to the third time, the corresponding error of the equipment's fault monitoring method was 0.00005. By comparison, the method proposed in the experiment had a smaller system error and higher convergence accuracy, which can quickly reach the target state and start working. Under the above experimental conditions, this paper randomly selected several groups of fault monitoring modeling variables, which are the sorting device data in normal and fault operation state. Wherein, A1 and A2 were the monitoring data of local burn through fault. A3 and A4 were the monitoring data of un-smooth slag discharge fault of the sorting device. A5 and A6 were the monitoring data of coking fault. In this paper, under three fault states of the sorting device, training was carried out in the fault monitoring models of the traditional and the domestic waste incineration slag sorting devices in this paper. A4 was the number of training groups. A5 was the processed data. The specific experimental results are shown in Table 4 below.

**Table 4:** Experimental results.

Method	Status	Localized burn through		Poor slag discharge		Coking	
		A1/°C	A2/°C	A3/*102 °C	A4	A5/%	A6/°C
Fault monitoring method for traditional domestic waste incineration slag sorting device	Normal operation status	154.775	183.708	1.464	13	0.095	143.879
		182.617	155.372	1.311	7	0.169	130.453
		185.542	139.188	1.273	13	0.078	142.406
	Fault operation status	652.708	538.337	1.883	40	0.396	357.783
		658.017	536.654	2.204	40	0.402	361.813
		628.008	514.200	1.834	43	0.405	402.297
The fault monitoring method of domestic waste incineration slag sorting device designed in this paper	Normal operation status	154.776	183.710	1.465	13	0.096	143.880
		182.630	155.373	1.310	7	0.170	130.450
		185.545	139.190	1.270	13	0.075	142.403
	Fault operation status	627.142	499.633	2.210	36	0.399	361.038
		616.525	504.087	2.285	35	0.395	360.817
		619.992	531.696	2.467	35	0.388	398.721

**Fig. 6:**
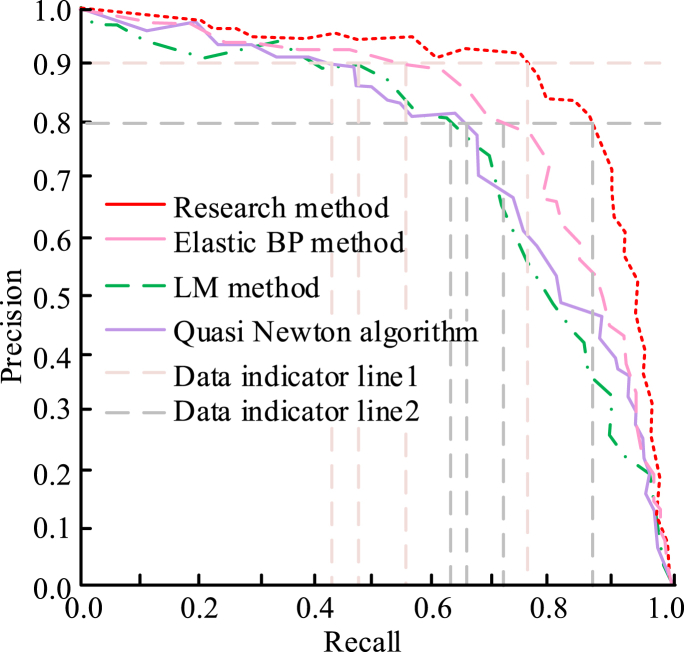
The PR curve changes for the CWRU dataset.

As shown in Tables 4 and in this experiment, three typical faults of incinerator slag sorting device, such as local burn through, unsmooth slag discharge and coking, were monitored in this paper. Among 100 training sample data, normal data: fault data = 7:3. Several groups of data were randomly selected to monitor the fault data. Since the domestic waste incineration slag sorting device mainly judged the fault condition by temperature, this paper analyzed the operation status of the sorting device according to the temperature monitoring data. In the process of monitoring fault data of sorting equipment, traditional methods had significant monitoring errors that affect monitoring accuracy. This proved that using the fault monitoring method of the traditional domestic waste incineration slag sorting device can only monitor the normal operation data, and the fault operation data monitoring effect is poor, which needs further improvement. However, after the training of the fault monitoring model of the domestic waste incineration slag sorting device designed in this paper, the monitoring accuracy was high, whether it was normal data or fault data. This proved that the fault monitoring method of the domestic waste incineration slag sorting device designed in this paper can accurately monitor the sorting device under normal operation and abnormal operation, which plays an important role in the incineration and sorting of domestic waste and meets the research purpose of this paper. Then the CWRU data set was selected as the main experimental data set, and the PR curve was used to evaluate the performance of the model constructed in the experiment. The specific results are shown in Fig. 6.

From the changes in the PR curve in Fig. 6, when the precision rates of the research methods were 80% and 90%, respectively, the corresponding recall rates were 0.887 and 0.724, respectively. At this time, the recall rates of the Elastic BP method were 0.718 and 0.584, respectively. At the same time, when the precision rate was 0.90, the recall rates of the LM algorithm and the Quasi-Newton method were 0.437 and 483, respectively. The above results all showed that under the same experimental conditions, the precision rate and recall rate of the research method have always been significantly higher than the other three methods. The higher precision rate and recall rate were the guarantee for the effectiveness of fault detection by the method constructed in the experiment. The experimental method could detect the CWRU data set, which directly proved that the constructed model has certain universality and generalization ability, and can be widely used in other fault detection fields.

## 4 Conclusion

The experiment proposed a fault monitoring method for domestic waste incineration slag sorting equipment based on BP neural network. In the process, the variables for constructing the fault monitoring model of the sorting device were first selected relatively accurately. Then the BP neural network was introduced to construct the fault monitoring model, and the effectiveness of the constructed model was verified through a series of experiments and data analysis. Controlling the power grid of the incinerator found that the temperature of the incinerator directly affects the temperature of the sorting device. With the iterative changes of the system, the corresponding monitoring errors under the operation of the steepest descent method, momentum BP method, adaptive learning rate BP method, elastic BP method, Quasi-Newton method and LM algorithm were respectively: 0.00225, 0.00372, 0.00099, 0.00028, 0.00024 and 0.00021. At the same time, when the system iterated to the third time, the corresponding error of the equipment fault monitoring method was 0.00005. The above results all showed that the research method has high convergence accuracy and operating efficiency, and can provide a more advanced and reliable fault monitoring method for domestic waste incineration slag sorting equipment. Despite the excellent performance of the experimental model, there are still some open issues and directions for future research. First, the current model may have limitations in identifying complex or rare failure modes. Second, in addition to fault monitoring, predicting potential failures and taking preventive measures are also an area worth exploring, and these are all research contents worth exploring in the future.

## CRediT authorship contribution statement

**Hao Xu:** Writing – original draft, Resources. **Dongdong Huan:** Methodology, Investigation. **Jihong Lin:** Writing – review & editing, Data curation.

## Declaration of competing interest

The authors declare that they have no known competing financial interests or personal relationships that could have appeared to influence the work reported in this paper.
